# Exploring perinatal ketamine for postpartum depression following cesarean section: A systematic review

**DOI:** 10.1002/pcn5.70004

**Published:** 2024-08-29

**Authors:** Jaylyn Thompson, David F. Lo, Alexis Foschini, Suvan Sundaresh

**Affiliations:** ^1^ Department of Medicine Rowan‐Virtua School of Osteopathic Medicine Stratford New Jersey USA; ^2^ Department of Research American Preventive Screening & Education Association (APSEA) Stratford New Jersey USA; ^3^ Department of Medicine Rutgers, The State University of New Jersey New Brunswick New Jersey USA; ^4^ Department of Research Lumina Institute 501(c)3 Cream Ridge New Jersey USA; ^5^ Department of Research Futures Forward Research Institute Toms River New Jersey USA; ^6^ Department of Research Penn State University State College Pennsylvania USA

**Keywords:** cesarean section, perinatal ketamine, postpartum depression

## Abstract

The aim of this study was to explore the use of perinatal ketamine to see if it can be used for the reduction of postpartum depression (PPD) following cesarean section (C‐section). PubMed, Cochrane, and Web of Science were the primary databases used for this review. Search terms used on January 5, 2024 incorporated “ketamine,” “C‐section,” “postpartum depression,” and related synonyms. The criteria for inclusion centered on studies published between January 1, 2008 and January 5, 2024. The final selection of articles was screened based on extraction criteria leaving eight randomized control trials in the final review. The selected data from the studies incorporated sample characteristics, study and population characteristics, and quantitative analyses covering Edinburgh Postpartum Depression Scale (EPDS) scores and depression rates. The Risk of Bias assessment was utilized to gain a deeper understanding of the quality of methodology used by the research studies. The review showed that ketamine can reduce the symptoms of PPD in mothers who have recently undergone C‐sections. Some studies showed decreased EPDS scores following the administration of ketamine while two studies also reported no significant differences in PPD following ketamine administration in C‐section patients. For example, Ma et al. found that the EPDS score at postpartum day 4 was significantly lower in the ketamine group compared with the control group (*p* = 0.007) while Yang et al. found that there were no significant differences between the ketamine and control group at 3 days postpartum (*p* = 0.553). The research from this review suggests that ketamine administration can prevent or decrease the symptoms of PPD, but more research is needed to establish the causal relationship between ketamine dosage and PPD in C‐section patients.

## INTRODUCTION

Postpartum depression (PPD) is defined as a major depressive episode that occurs in the perinatal period or with the presence of mood symptoms occurring in the 4 weeks following childbirth. PPD symptoms include crying spells, intense irritability, difficulty bonding, and more. Depression is the most common complication of childbirth with a robust presence of 10–15%.^1^ PPD has adverse effects not only on mothers but also on children and spouses. With this, it is plausible to consider that PPD leads to a social and emotional disconnect within families, being termed the second greatest cause of global disease burden by the World Health Organization.^1^, ^2^ The topic of PPD is multifaceted as the condition can occur in primiparous and multiparous women.^3^ Aside from the emotional implications of PPD, one in eight women will continue to struggle at the 2‐year mark following childbirth, leading to financial implications. The immense financial burden to society is estimated to amount to $2.8 billion annually, due to the reduced economic productivity among mothers and additional perinatal issues with subsequent pregnancies.^4^

To concisely analyze the use of a new compound for the reduction of PPD, one must analyze what the current medication management entails. For all mothers, there should be an emphasis on self‐care, good sleep habits, exercise, and the use of any psychosocial factors that may be available.^5^ Management is solely dependent on the severity of PPD in birthing people. First‐line treatment includes nonpharmacologic options, which most commonly consist of cognitive‐behavioral therapy, interpersonal therapy, and other forms of supportive counseling.^6^ If patients experience an insufficient response to this, the next option would include pharmacologic management. Selective serotonin reuptake inhibitors (SSRIs) are the most commonly used. While using medical management it is important to consider the pharmacologic passage of some SSRIs, most commonly sertraline, through breast milk. Finally, one may also consider somatic therapies, such as electroconvulsive therapy, which may be used in the setting of psychosis or suicidality.^5^

**Figure 1:**
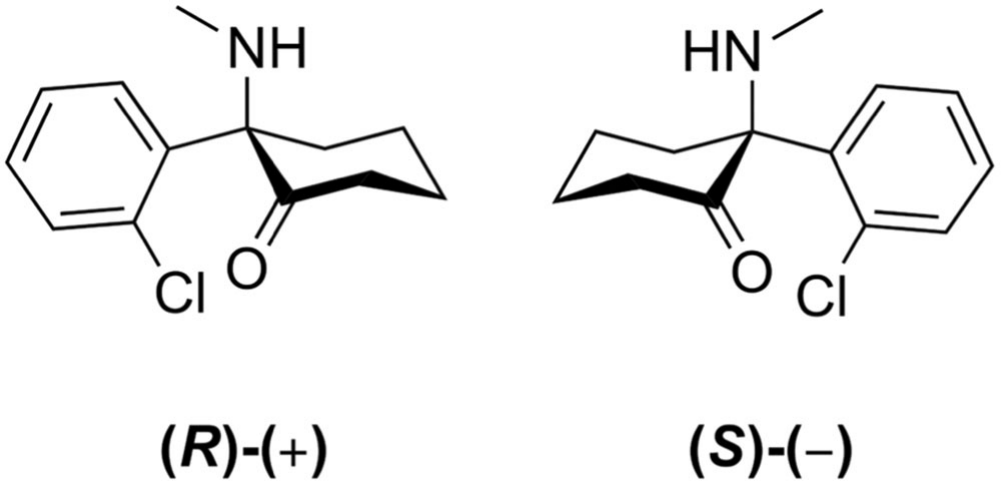
Structural formulas for S‐ and R‐ketamine.

PPD following cesarean section (C‐section) is a significant factor that appears in over 30% of women who undergo C‐section. With 29% of all births by 2030 expected to be C‐sections, it is imperative to address alternative treatment for PPD.^1^ The use of ketamine to combat PPD is important, as severe depression is more likely to occur perinatally than at any other point for mothers.^7^ Ketamine is a noncompetitive antagonist of *N*‐methyl‐d‐aspartate. It has the potential for use as an anesthetic and may have reduction effects on PPD, as shown in Figure 1.^8^, ^9^

Ketamine is thought to increase brain‐derived neurotrophic factor levels, which may produce a rapid onset of antidepressant action.^10^ Our paper evaluates the efficacy of ketamine as an adjuvant anesthetic that reduces PPD. Ketamine is water‐ and lipid‐soluble and can be administered intravenously, intramuscularly, intranasally, and orally. It has been used in operating rooms for over 50 years. Its predominant use is for unstable hemodynamics, burns, and procedures in children.^8^ Ketamine is found in two isomers: the S(+) isomer and the R(−) isomer.^11^ As an anesthetic, ketamine is known to have psychoactive, memory, cognitive, and peripheral effects as well as abuse potential. Most, if not all, side‐effects of ketamine depend on the dose, are transient, and are known to be self‐resolving.^12^

Several studies have delved into the use of ketamine in the treatment of PPD.^2^, ^8^, ^9^, ^11^, ^12^ Despite that, there is limited research specifically investigating the S‐isomer of ketamine and various methods of ketamine administration for the treatment of PPD. The existing literature presents discrepancies in methodologies across studies and variations in sample sizes contribute to this inconsistency. For example, in their 2023 review, Liu et al. recognize the emerging literature showing evidence that S‐Ketamine as an adjuvant reduces PPD. Their randomized control trial (RCT), however, showed no evidence of its effectiveness for women undergoing elective C‐section. However, this article was limited by its low sample size and lack of structured diagnoses for PPD. The findings did show a reduced risk of PPD in the S‐ketamine group when compared to the control group, though these results were not statistically significant. A larger sample size may emphasize these differences and add more statistical significance. Furthermore, medical record reviews and proper diagnoses were not used to assess PPD risk. The EPDS screening was used as a substitute, which may be insufficient holistically. EPDS scores were also shown to be lowered in the S‐ketamine group, but were not statistically significant which once again may be due to the low sample size of the study.^13^ The lack of consensus impedes the establishment of ketamine as a definitive therapeutic strategy for PPD. Our paper is unique as it acknowledges the need for careful consideration of ketamine as a treatment modality for PPD. This research is a vital stepping stone as well as a multidisciplinary approach to treating a condition that affects millions of mothers worldwide. Addressing this knowledge gap is imperative for advancing ketamine use as a viable approach in the management of PPD. Our paper is unique in that it explores medical management with a distinct method of action that may have a more rapid onset of relief and a longer duration of action for mothers. We are viewing ketamine as an unconventional approach that may allow for its incorporation into a more comprehensive treatment plan for mothers battling PPD.

## METHODS

**Figure 2:**
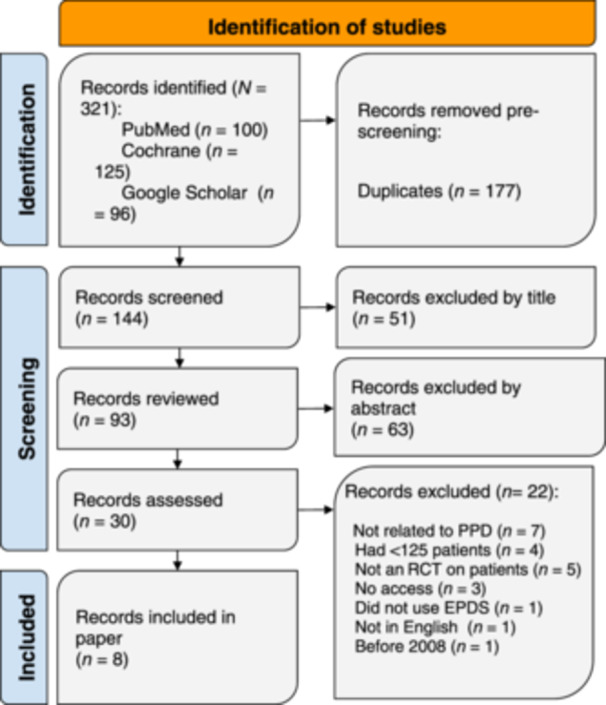
PRISMA flowchart illustrating the systematic review process for the use of ketamine for postpartum depression (PPD) after cesarean section. The flowchart outlines the selection and screening of studies, including search strategies, eligibility criteria, and the final inclusion of relevant studies for data synthesis and analysis. RCT, randomized control trial.

This systematic review meticulously followed the Preferred Reporting Items for Systematic Reviews and Meta‐Analyses (PRISMA) guidelines; however, limited clinical studies availability rendered a meta‐analysis impractical. The procedures of this review were carried out in strict accordance with the 2015 PRISMA recommendations as shown in Figure 2.^14^

### Search methodology

This paper is a structured review of the current literature that is used to describe and understand the relationship between the use of perinatal ketamine on PPD. We conducted a comprehensive systematic search across multiple databases, including PubMed, Cochrane Library, and Web of Science. This search included papers within the last 15 years, encompassing the period from March 2017 until September 2023, and it was guided by a search strategy featuring specific MeSH terms, such as “Ketamine” AND “Postpartum” AND “Depression” AND “Cesarean Section,” as shown in Table 1.

**Table 1:** MeSH search term strategy

PubMed	(“Ketamine” OR “NMDA receptor antagonist” OR “NMDA” OR “Ketalar” OR “Calipsol” OR “Esketamine” OR “Anesthetic” OR “Anesthesia” OR “Tranquilizer” OR “Hallucinogen”) AND (“Postpartum” OR “Postpartum Period” OR “Puerperium” OR “Perinatal” OR “After birth”) AND (“Depression” OR “MDD” OR “Major Depressive Disorder” OR “Depressive”) AND (“C‐Section” OR “Cesarean” OR “Caesarian” OR “Post Cesarean” OR “Cesarean Delivery” OR “Cesarean Section” OR “Abdominal” OR “Abdominal Delivery”)
Cochrane	(“Ketamine” OR “NMDA receptor antagonist” OR “NMDA” OR “Ketalar” OR “Calipsol” OR “Esketamine” OR “Anesthetic” OR “Anesthesia” OR “Tranquilizer” OR “Hallucinogen”) AND (“Postpartum” OR “Postpartum Period” OR “Puerperium” OR “Perinatal” OR “After birth”) AND (“Depression” OR “MDD” OR “Major Depressive Disorder” OR “Depressive”) AND (“C‐Section” OR “Cesarean” OR “Caesarian” OR “Post Cesarean” OR “Cesarean Delivery” OR “Cesarean Section” OR “Abdominal” OR “Abdominal Delivery”)
Web of Science	(“Ketamine” OR “NMDA receptor antagonist” OR “NMDA” OR “Ketalar” OR “Calipsol” OR “Esketamine” OR “Anesthetic” OR “Anesthesia” OR “Tranquilizer” OR “Hallucinogen”) AND (“Postpartum” OR “Postpartum Period” OR “Puerperium” OR “Perinatal” OR “After birth”) AND (“Depression” OR “MDD” OR “Major Depressive Disorder” OR “Depressive”) AND (“C‐Section” OR “Cesarean” OR “Caesarian” OR “Post Cesarean” OR “Cesarean Delivery” OR “Cesarean Section” OR “Abdominal” OR “Abdominal Delivery”)

During our exploration of databases, we scrutinized material that was electronic as well as a manual review of the bibliographic studies included. In addition, we took a deeper look into book chapters and original articles from our bibliographic studies to better supplement our primary research. Succeeding this, two of our reviewers used a standardized approach to progress to the selection phase (J.T., A.F.). These reviewers then independently completed a meticulous evaluation of potential studies, as well as the study's full text.

### Study selection

By following a comprehensive two‐tried screening approach, a thorough review was conducted on the titles, abstracts, and relevant articles. Inconsistencies were strictly reviewed and resolved through discussion. Any publications in question progressed to the full‐text review phase. Later, the full texts of articles that successfully passed the first screening were subject to an in‐depth analysis, by both independent reviewers. Disparities that remained after this stage were exhaustively analyzed by the two reviewers. If necessary, a third reviewer was engaged to resolve any disagreements (D.L.).

### Data extraction

The timeframe in which data were obtained was from the last 15 years. The data included things like RCTs and quantitative and qualitative studies. An initial database was created and extracted independently by two researchers (J.T. and A.F.). Data were fully assessed for consistency in outcomes. Any discrepancies that arose were resolved via discussion and consensus between these two researchers.

### Inclusion criteria

In this systematic review, the relationship between ketamine as a perinatal anesthetic for the reduction of PPD was reviewed. Selection criteria were developed to determine the quality and relevance of the research reviewed. In the selection process, RCTs were preferentially used. Additionally, we prioritized RCTs that used the Edinburgh Postpartum Depression Scale (EPDS) to assess perceived depression in the perinatal period. Furthermore, it was required that patients had had a C‐section as a method of delivery to control for the anesthetic used. Priority was given to RCTs that used intravenous (iv) infusion of ketamine to ensure standardization in care. Moreover, papers were not limited to a geographic location. Study types as a whole consisted of RCTs, literature reviews (used only for background information), and one meta‐analysis. The literature reviews and meta‐analysis included preferentially reviewing the impact of ketamine as an adjuvant to anesthesia when women had undergone planned C‐section. These stringent criteria were essential in guaranteeing direct relevance in the use of ketamine to view its effects on PPD.

### Exclusion criteria

To refine the research selection process, exclusion criteria were carefully implemented. In our selected RCTs, we eliminated studies that did not include sufficient patient data as this would cause limitations in the reputability. We implemented restrictions on the use of English as the sole language of the study. To avoid bias being incorporated into the studies, we eliminated studies that had flaws in their design, analysis, or data collection. Studies were limited to a sample size of greater than 130 human participants. Studies not completed in the last 15 years or not related to ketamine and PPD were excluded. These exclusion criteria allowed us to best assess the use of ketamine during C‐sections and its impact on PPD.

### Risk of bias

Succeeding the removal of duplicates, items were assessed for relevance based on title and abstract. Full‐text appraisal and risk of bias were conducted by two reviewers (J.T. and A.F.) on studies deemed potentially eligible using the updated 2008 Cochrane Risk of Bias 2 (RoB2) Scale for RCTs. We used specific criteria (low, some concerns, high) to classify the risk of bias, as shown in Table 2. In case of debate, a third reviewer (D.L.) made the final determination; however, this event was not necessary in the case of this systematic review. Eligible studies were then subjected to data extraction.

**Table 2:** Risk of bias assessment^15^

Author	Bias from randomization process	Bias from intervention deviations	Bias from missing outcome data	Bias in measurement of outcomes	Bias in selection of reported result
Alipoor et al.^1^	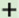	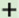	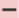	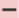	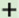
Han et al.^7^	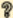	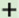	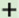	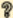	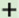
Yao et al.^10^	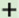	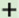	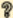	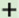	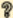
Wang W et al.^3^	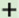	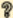	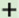	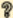	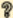
Wang Y et al.^16^	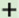	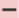	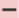	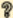	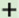
Ma et al.^17^	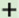	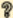	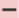	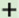	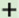
Xu et al.^18^	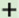	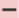	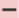	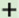	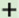
Yang et al.^19^	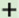	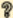	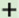	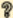	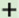

Abbreviations: 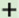, low risk of bias; 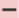, high risk of bias; 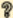, some concerns.

### Data analysis

The data extracted were analyzed, reviewed, and conducted to discuss the impacts of ketamine on PPD. The EPDS was used during the studies intermittently as a 10‐option questionnaire used to assess depression outcomes.

## RESULTS

After a thorough systematic review, a total of eight RCTs met the established inclusion criteria and became part of the comprehensive systematic review. Among these, one occurred in Iran and the rest in China. The cumulative studies encompassed 2387 birthing people, with individual study samples ranging from 134 to 654 participants. The focal demographic across all the included publications was a specific population aged 18 to 45 years, all of whom were scheduled to undergo C‐section for delivery. A condensed overview of the incorporated studies can be found in Table 3.

**Table 3:** Sample characteristics

Author	Country	Year	No. of initial subjects	No. excluded	No. of final subjects	EPDS	Duration of study
Alipoor et al.^1^	Iran	2020	134	0	134	Yes	NR
Han et al.^7^	China	2022	451	176	275	Yes	09/2019–07/2020
Yao et al.^10^	China	2020	502	194	308	Yes	01/2019–07/2019
Wang W et al.^3^	China	2022	160	4	156	Yes	05/2021–12/2021
Wang Y et al.^16^	China	2022	1138	898	240	Yes	03/2018–02/2020
Ma et al.^17^	China	2019	702	48	654	Yes	09/2014–12/2016
Xu et al.^18^	China	2017	483	158	325	Yes	10/2015–03/2016
Yang et al.^19^	China	2020	312	17	295	Yes	12/2020–01/2022

Abbreviations: EPDS, Edinburgh Postpartum Depression Scale; NR, not reported.

Alipoor et al. included 134 Iranian pregnant mothers ranging from 18 to 35 years old that were eligible for C‐sections in the year 2020.^1^ In the same year, Yao et al. examined 502 participants with 194 leaving the study prematurely.^10^ Han et al. carried out a comprehensive study in China in 2022, which included 451 initial subjects in 2019.^7^ Notably, 176 subjects discontinued their involvement in the study. In the study by W. Wang et al. in 2022 in China, 160 subjects were observed, and only four participants dropped out, making it a relatively stable study.^3^ The study by Y. Wang et al., also conducted in China in 2022, had a larger starting cohort of 1138 subjects.^16^ A total of 898 participants were excluded during this study. Ma et al. also conducted a large‐scale study in China in 2019 with 702 starting subjects, where 48 participants withdrew from the research, introducing some variance in the data.^17^ Furthermore, Xu et al. observed 483 starting subjects scheduled to undergo C‐sections with 158 participants dropping out.^18^ Finally, Yang et al. conducted their research in China in 2020. They began the study with 312 participants, but 17 were excluded, leaving 295 subjects.^19^ Collectively, the diverse studies presented here promote variance not only in terms of the countries of origin but also in the number of subjects, dropout rates, and the duration of the research.

The interventions across the studies involved a spectrum of ketamine dosages and administration methods, summarized in Table 4. These included varied ketamine concentrations, combinations with other medications, and distinct routes of administration, highlighting the multifaceted approaches investigated in the context of PPD. Alipoor et al. utilized a combination of Nesdonal and ketamine.^1^ Yao et al. and Xu et al. used ketamine diluted with 0.9% saline, while Han et al., Y. Wang et al., and Yang et al. implemented esketamine administration.^7^, ^10^, ^16^, ^18^, ^19^ W. Wang et al. implemented multiple dosage groups ranging from high to low ketamine intervention.^3^ Lastly, Ma et al. incorporated specific combinations of ketamine, sufentanil, and palonosetron hydrochloride.^17^ Patient ages across studies varied, with the majority falling within the range of 18 to 45 years. Gestational ages at delivery also showed diversity, spanning from 36 to 42 weeks. While some studies reported specific age and gestational age ranges, others provided only general categories or did not disclose this information. Different studies utilized distinct time intervals for EPDS evaluations, with assessment points ranging from 0 to 90 days post‐C‐section. This variability enhances the overall understanding of ketamine interventions over different time frames.

**Table 4:** Study and population characteristics

Author	Intervention group		Control group	Age of patients (years)	Average study gestational age (weeks)	EPDS (days post C‐sx)
	Ketamine	Adjuvant				
Alipoor et al.^1^	0.5 mg/kg	1–2 mg/kg Nesdonal	3–5 mg/kg Nesdonal	18–35	NR	0, 14, 28
Han et al.^7^	0.5 mg/kg S‐ketamine	2 μg/kg sufentanil + 10 mg tropisetron	2 μg/kg sufentanil + 10 mg tropisetron	18–45	36–42	3, 14, 28
Yao et al.^10^	0.25 mg/kg diluted to 5 mL	0.9% saline	5 mL 0.9% saline	26–34 (IG) 27–33 (CG)	37–42	7, 14, 30
Wang W et al.^3^	Grp H: 0.4 mg/kg Grp M: 0.2 mg/kg Grp L: 0.1 mg/kg	NR	1.5 μg/kg sufentanil + 4 mg totanisoltron, diluted to 150 mL with saline	22–35	Grp H: 39.1 Grp M: 39.3 Grp L: 39.5 CG: 39.4	7, 42
Wang Y et al.^16^	0.2–0.5 mg/kg S‐ketamine	50 µg sufentanil citrate, 0.25 mg palonosetron HCl in 200 mL saline	50 µg sufentanil citrate, 0.25 mg palonosetron HCl in 200 mL saline	25–34	NR	0,7, 42, 90
Ma et al.^17^	160 mg	100 μg sufentanil and 0.25 mg palonosetron HCl in 100 mL saline	100 μg sufentanil and 0.25 mg palonosetron HCl in 100 mL saline	>18	NR	0, 42–56
Xu et al.^18^	0.25 mg/kg diluted to 10 mL	0.9% saline	10 mL of 0.9% saline	IG: 27–35 CG: 28–36	IG: 37.4–40.6 CG:37.6–40.1	3, 42
Yang et al.^19^	IG1: 1 mg/kg S‐ketamine PCIA IG2: 2 mg/kg S‐ketamine PCIA	NR	20 ml of 0.9% normal saline by infusion pump over 10 min	IG1: 31.7 IG2: 31.9 CG: 32.2	IG1: 38.5 IG2: 38.6 CG: 38.5	7, 42

Abbreviations: CG, control group; C‐sx, cesarean section; EPDS, Edinburgh Postpartum Depression Scale; Group C, control group (sufentanil 1.5 ug/kg + totanisoltron 4 mg); Grp H, high‐dose group (0.4 mg/kg); Grp L, low‐dose group (0.1 mg/kg); Grp M, middle‐dose group (0.2 mg/kg); HCl, hydrochloride; IG, intervention group; iv, intravenous; Nesdonal, sodium thiopental; NR, not reported; PCIA, patient‐controlled intravenous analgesia.

Overall, six out of eight papers predominantly found that iv ketamine during cesarean delivery effectively prevents PPD from 3 days to 1 month, as shown in Table 5. Additionally, the use of ketamine could result in a reduction of the utilization of morphine in the perinatal period and promote an overall greater quality of recovery. For safety considerations, due to the known adverse effects of ketamine, low‐dose ketamine may be more suitable for pregnant women having a C‐section.^16^ Using an original pregnancy stress scale, one study found that ketamine may work better for moderate stress levels rather than mild and severe stress levels. Factors that influence this scale include education, occupation, and socioeconomic status. It is unknown why this occurred, and more research is recommended to be completed on this topic.^17^ Finally, one study found no significant differences in PPD between the two groups at 3 days and 6 weeks after delivery.^18^

**Table 5:** Study results

Author	Results	Conclusion	Effect of ketamine
Alipoor et al.^1^	Patients in the ketamine group compared to the control group had significantly lowered (*p* < 0.0001) EPDS scores 2 weeks after (11.82 ± 3.41 vs.14.34 ± 4.29, respectively) and 4 weeks after (10.84 ± 3.48 vs.13.09 ± 3.79) cesarean delivery.	Ketamine is effective in preventing PPD 2 and 4 weeks after cesarean delivery.	+
Han et al.^7^	On postoperative days 3, 14, and 28, depression rates were significantly lower (*p* < 0.05) in the S group (8.2%, 9.8%, and 17.2%) compared to the C group (17.6%, 24.2%, and 19.0%) except for day 28 (*p* = 0.76).	Addition of S‐ketamine (0.01 mg/kg/h) to PCIA may decrease the occurrence of PPD within 14 days.	∅
Yao et al.^10^	At 1 week postpartum, there were significant differences between the ketamine and placebo groups (13.1% vs. 22.6%, respectively; *p* = 0.029). No significant difference was found at 2 weeks (11.8% vs. 16.8%, respectively; *p* = 0.209) and 1 month postpartum (10.5% vs. 14.2%, respectively; *p* = 0.319).	Intravenous ketamine (0.25 mg/kg) during surgery may alleviate postpartum depressive symptoms for 1 week, with the long‐term effects yet to be determined.	+
Wang W et al.^3^	Group H had significantly lowered incidences of PPD at 1 week and 6 weeks post‐delivery than Group M, Group L, and Group C (*p* < 0.01). Group M had significantly lower incidences of PPD than Group L and Group C (*p* < 0.01), and Group L significantly lower than Group C (*p* < 0.01).	Esketamine with sufentanil in post‐C‐section patient‐controlled analgesia can lower PPD rates at 1 and 6 weeks after delivery.	+
Wang Y et al.^16^	Patients in the esketamine group had significantly lowered incidences of PPD (*p* = 0.002).	Esketamine reduces the incidence of PPD within 3 months of cesarean delivery.	+
Ma et al.^17^	PPD prevalence in the ketamine group when compared to the control group (12.8% vs. 19.6%, respectively) was significantly lower (*p* = 0.020) than in the control group.	Ketamine administration can reduce depressive symptoms in the early postpartum period and is a preventative measure for PPD.	+
Xu et al.^18^	At 3 days postpartum, there were no significant differences between the ketamine and control group on PPD (25.3% vs. 28.2%, respectively; *p* = 0.553). At 6 weeks postpartum, no significant differences were found on PPD once again (16.0% vs. 17.8%; *p* = 0.675).	The use of ketamine on post‐C‐section patients did not have any significant differences in PPD at 3 days and 6 weeks postpartum.	∅
Yang et al.^19^	Esketamine (0.25 mg/kg) followed by PCIA of esketamine (1 mg/kg) reduces PDS incidence at 7 days postpartum. Esketamine (0.25 mg/kg) followed by PCIA of ketamine (2 mg/kg) reduces PDS incidence at 7 and 42 days postpartum.	This study found that a loading dose of esketamine (0.25 mg/kg by micropump) combined with a continuous esketamine PCIA could be a safe and effective way to reduce PDS.	+

Abbreviations: +, positive; ∅, neutral; PCIA, patient‐controlled intravenous analgesia; PDS, postpartum depressive symptoms; PPD, postpartum depression.

## DISCUSSION

### Clinical implications

Ketamine has demonstrated potential clinical implications for the management of PPD symptoms. Notably, the significant reduction in PPD observed in mothers who received iv S‐ketamine during C‐section suggests a promising avenue for management. When recommending ketamine interventions, clinicians should consider the specific isomer and method of administration. While the evidence suggests the benefits of S‐ketamine administration during C‐section, further research is needed to understand its impact on symptoms of PPD.

Additionally, S‐ketamine has been shown to reduce symptoms of PPD significantly. In four of the studies, iv injection of ketamine was found to reduce rates of PPD specifically at days 3 and 14 post‐C‐section.^1^, ^3^, ^10^, ^18^ These four studies all maintained similar ages (18–25 years), weights (50–100 kg), dosages (0.25 mg/kg), and methods of administration (iv).^3^, ^10^ However, one study found no statistical significance in the prevalence of PPD 3 days or 6 months after C‐section and intraoperative ketamine infusion.^18^

Research suggests that ketamine has antidepressive and analgesic effects, a short infusion time, and a rapid clearance.^2^ A specific dosage, timeframe, and administration method of ketamine to efficiently treat PPD is currently unknown as conflicting research currently exists. One study found that an analgesic pump given for 48 h postpartum may show superiority in PPD prevention in comparison to iv injection during C‐section.^20^ Further research should be done on different administration methods of ketamine to maximize its therapeutic effects.

While the mechanism by which ketamine reduces PPD is not well understood, newer research has delved into the theory of neuroplasticity and its influence on depression. Neuroplasticity, or the neuronal adaptations the brain uses to recognize itself when facing internal or external stimuli, is a promising area of future research. Major depressive disorder (MDD) has shown a strong connection to abnormalities in neuroplasticity and the impact of ketamine on this is a pathway that needs to be further explored.^21^

Given the multifaceted nature of PPD symptoms, a multidisciplinary approach is required to give optimal care. A multidisciplinary approach that combines ketamine with other therapeutic modalities, such as cognitive‐based therapy (CBT), may yield comprehensive benefits for the patients. Clinicians should explore integrated treatment plans tailored to individual patient needs, considering the potential synergistic effects of combining ketamine administration of CBT to treat PPD.

### Clinical highlights

- In evaluating the use of S‐ketamine as an adjuvant in reducing the incidence of PPD during C‐sections, several key clinical highlights were observed: S‐ketamine as an adjuvant reduces the incidence of PPD.^1^, ^3^, ^4^, ^7^, ^10^, ^16^, ^17^
- Due to the known adverse effects of ketamine, low‐dose ketamine may be more suitable for use during a C‐section.^16^
- Adverse effects with a statistically significant occurrence include headache, hallucinations, and dizziness.^10^
- More research is needed on the different protocols and integration techniques of ketamine used to improve its efficacy.

### Limitations

This study has limitations that should be considered when interpreting the results. First, all patients had C‐sections, which limits the provision of information about ketamine treating PPD in other birth models. Future studies should aim to include a more diverse patient population to better understand the effects of different birth models and the likelihood of PPD. Second, it is difficult to estimate the influence of various administrative methods of ketamine on the effects of PPD. Future studies should consider comparing the modes of administration and the effects of PPD symptoms. Third, the studies were all published in English, which may limit the ability to generalize the findings to research published in other languages. Further research should aim to include studies in other languages to increase the diversity of the literature review. Lastly, most studies had a small sample size, which may limit greater generalizability of the findings. Studies should aim to include larger sample sizes to increase the statistical power and validity of the results. One study by Ma et al. also did not administer ketamine by weight, which could be a limitation, as all the other studies did. Therefore, more research on the effects of ketamine in larger population sizes, in different demographic populations, and at varying time intervals pre‐, peri‐, and postpartum needs to be performed.

### Future directions

While the studies included in this systematic review primarily focused on the iv administration of ketamine, it is crucial to extend these investigations to other methods of administration. By doing so, researchers can provide a more comprehensive understanding of how patients with PPD could be treated at home or with less medical intervention, hopefully decreasing noncompliance issues. Another critical avenue for future research is the effects of ketamine on the growing fetus. This nuanced perspective will be instrumental in tailoring treatment strategies to individual patient needs. To enhance the statistical power and reliability of results, future studies should aim for larger sample sizes. This will allow for more robust conclusions as to the effectiveness of ketamine in treating PPD and provide stronger evidence for its clinical use.

## CONCLUSION

In conclusion, PPD is the most common complication of pregnancy and it has been shown to have destructive effects not only on the mother, but on the neonate, family, and society.^1^ Overall, it can be concluded that S‐ketamine as an adjuvant reduces the incidence of PPD and relieves pain 48 h after delivery without significant adverse effects.^7^ Despite the incidences of lowered PPD upon S‐ketamine administration, authors of the various studies used also convey the need for more research on the optimal doses, administration methods, and timing for S‐ketamine application. Due to the sensitive nature of the topic, limited research exists on why C‐sections are the leading mode of delivery to cause PPD. Additional research needs to be conducted on the side‐effects of ketamine as an anesthetic and its effects on the fetus, such as the ability to cross the placenta. Furthermore, with the emotional impact of the COVID‐19 pandemic worldwide, rates of PPD have increased drastically. Hence, research navigating ketamine usage in PPD is even more important to assess.

## AUTHOR CONTRIBUTIONS

Jaylyn Thompson wrote the initial manuscript and came up with the idea for the paper. David F. Lo, Alexis Foschini, and Suvan Sundaresh edited, reviewed, and added to the manuscript. All authors approved the final version of the manuscript.

## CONFLICT OF INTEREST STATEMENT

The authors declare no conflicts of interest.

## ETHICS APPROVAL STATEMENT

The protocol for this research project has been approved by the suitably constituted ethics committee of Rutgers University, and it does conform to the provisions of the Declaration of Helsinki.

## PATIENT CONSENT STATEMENT

All patients in the studies gave informed consent and their anonymity was preserved.

## CLINICAL TRIAL REGISTRATION

This study is a systematic review and does not involve any clinical trials; therefore, clinical trial registration was not required.

## Data Availability

The data supporting the findings of this study are available upon reasonable request from the corresponding author.
